# Supplementary material for: FluoMALDI Microscopy: Matrix Co‐Crystallization Simultaneously Enhances Fluorescence and MALDI Imaging
Source: Adv Sci (Weinh). 2023 Oct 31;10(35):2304343. doi: 10.1002/advs.202304343 (PMC10724403; doi:10.1002/advs.202304343)
Supplement: Supplementary file 1 — Supporting Information [file ADVS-10-2304343-s003.pdf]

ADVANCED SCIENCE
Open Access

Supporting Information

for *Adv. Sci.*, DOI 10.1002/advs.202304343

FluoMALDI Microscopy: Matrix Co-Crystallization Simultaneously Enhances Fluorescence and MALDI Imaging

*Ethan Yang, Xinyi Elaine Shen, Hoku West-Foyle, Tae-Hun Hahm, Maxime A. Siegler, Dalton R. Brown, Cole C. Johnson, Jeong Hee Kim, LaToya Ann Roker, Caitlin M. Tressler, Ishan Barman, Scot C. Kuo and Kristine Glunde**

Supporting Information

FluoMALDI microscopy: matrix co-crystallization simultaneously enhances fluorescence and MALDI imaging

Ethan Yang[#,1,2], Xinyi Elaine Shen[#,1,2], Hoku West-Foyle[3,4], Tae-Hun Hahm[1,2], Maxime A. Siegler[5], Dalton R. Brown[1,2], Cole C. Johnson[1,2], Jeong Hee Kim[6], LaToya Ann Roker[3,4], Caitlin M. Tressler[1,2], Ishan Barman[1,6,7], Scot C. Kuo[3,4,8], Kristine Glunde*[,1,2,7,9]

1. Russel H. Morgan Department of Radiology and Radiological Science, Johns Hopkins University School of Medicine, Baltimore, MD, USA.
2. Applied Imaging Mass Spectrometry Core, Johns Hopkins University School of Medicine, Baltimore, MD, USA.
3. Microscope Facility, Johns Hopkins University School of Medicine, Baltimore, MD, USA.
4. Department of Cell Biology, Johns Hopkins University School of Medicine, Baltimore, MD, USA.
5. Department of Chemistry, Johns Hopkins University, Baltimore, MD, USA.
6. Department of Mechanical Engineering, Johns Hopkins University, Baltimore, MD, USA.
7. Sidney Kimmel Comprehensive Cancer Cancer, Johns Hopkins University School of Medicine, Baltimore, MD, USA.
8. Department of Biomedical Engineering, Johns Hopkins University School of Medicine, Baltimore, MD, USA.
9. Department of Biological Chemistry, Johns Hopkins University School of Medicine, Baltimore, MD, USA.

\# **These authors contributed equally to this work.**

*** Corresponding author**
Kristine Glunde, Ph.D.
Professor of Radiology, Oncology, and Biological Chemistry
Johns Hopkins University School of Medicine
Radiology Department - Division of Cancer Imaging Research
Traylor Building, Room 203
720 Rutland Avenue
Baltimore, Maryland 21205, U.S.A.
Phone: +1 410 614 2705
Fax: +1 410 614 1948
E-mail: kglunde1@jhmi.edu

Funding information
National Institutes of Health grants R01 CA213492, R01 CA213428, R01 CA264901, S10 OD030500, S10 OD030352.

Table of Contents for Supporting Information

Supporting Methods **3**
Supporting Methods: Fluorescence image processing and data analysis – additional details 3
Supporting Methods: MALDI mass spectrometry imaging – additional details 3
Supporting Methods: MALDI MSI data processing and analysis – additional details 4
Supporting Methods: Single-crystal X-ray Crystallography – additional details 4

Supporting Tables **7**
Supporting Table S1: Assignments of MALDI-MSI signals in mouse brain sections 7
Supporting Table S2: Effects of fluorescence slide scanning on MALDI imaging 8

Supporting Figures **9**
Supporting Figure S1: Tandem MS spectra of Rhodamine B and pink Sharpie® 9
Supporting Figure S2: FluoMALDI fluorescence enhancement of exogenous fluorophores 10
Supporting Figure S3: FluoMALDI fluorescence enhancement of endogenous fluorophores 11
Supporting Figure S4: FluoMALDI autofluorescence enhancement of mouse brain sections 12
Supporting Figure S5: FluoMALDI autofluorescence enhancement of mouse kidney sections 13
Supporting Figure S6: Tandem MS spectra of m/z^- 885.8 Da identified as PI (38:4) 14
Supporting Figure S7: Spectra, m/z images of CHCA-coated mouse brain halves from Fig. 3 15
Supporting Figure S8: Spectra, m/z images of 9AA-coated mouse brain halves from Fig. 3 16
Supporting Figure S9: Spectra, m/z images of nH-coated mouse brain halves from Fig. 3 17
Supporting Figure S10: Tandem MS spectra of m/z^+ 785.45 Da identified as PA (20:3_20:4) 18
Supporting Figure S11: Tandem MS spectra of m/z^- 621.30 Da identified as LPI (20:3) 19
Supporting Figure S12: Tandem MS spectra of m/z^- 906.63 Da identified as SHexCer (42:1);O3 20
Supporting Figure S13: Full length co-crystallization time course for ROD+CHCA 21
Supporting Figure S14: Single-crystal X-ray crystallography data of ROD+CHCA co-crystals 22
Supporting Figure S15: Brightfield and H-E-stained images of brain section in Fig. 6 23
Supporting Figure S16: FluoMALDI spectral imaging for all acquired excitation wavelengths 24
Supporting Figure S17: Tandem MS spectra of m/z^+ 478.33 Da identified as LPE (18:2) 25
Supporting Figure S18: Tandem MS spectra of m/z^+ 504.04 Da identified as PC (O-16:0) 26
Supporting Figure S19: Effects of fluorescence slide scanning on MALDI imaging 27

Author Contributions **28**

Supporting Methods

Fluorescence Image Processing and Data Analysis – Additional Details

For fluorescence measurements, we quantified the mean pixel fluorescence intensity (MPFI), which accounted for different matrices having different fluorescence backgrounds. For quantitative measurement of Sharpie ® J markings, we measured MPFI from the entire region of the J for each matrix, solvent, and control (none). MPFI background away from the J marking was measured as well. We measured the red fluorescence intensity on and off the J markings based on these MPFI values according to the following formula: $FL\ Intensity\ (a.u.)\ =\ MPFI(on-J) - MPFI(off-J)$.

To calculate the fold enhancement of fluorescence intensities from the J markings, we applied the following formula: $Fold\ Enhancement = \frac{MPFI(on-J\ matrix)/MPFI(off-J\ matrix)}{MPFI(on-J\ none)/MPFI(off-J\ none)}$.

To measure fold change for different matrix densities on Sharpie ® lines, we used five randomly selected regions, placed within the lines to avoid any edge effects, to quantify MPFI. These MPFI values were used in the following formula, which is given for the example of 8 layers (8x) of CHCA matrix compared to no matrix (0x): $Fold\ Change = \frac{FL\ Intensity(8x)}{FL\ Intensity\ (0x)}$.

For quantitative measurement of fluorophore spots, we measured the green and red fluorescence intensity on and off the fluorophore spots for CHCA coated spots and uncoated controls (none). Each fluorophore spot was circled based on the brightfield images at a diameter inside the spot that avoided the dried edge effect. Quantification of all tested fluorophores with CHCA matrix was performed according to the formula: $Fold\ Change = \frac{FL\ Intensity(CHCA)}{FL\ Intensity\ (none)}$.

For quantitative measurements of brain and kidney sections, we quantified MPFI on and off tissue on both the coated and uncoated halves of brains and kidneys. For on-tissue MPFI measurements, we circled the entire coated or uncoated half of the brain or kidney, or the entire uncoated brain or kidney for “none” and “solvent” controls and calculated the MPFI. For off tissue measurements, we chose five randomly selected regions off tissue to calculate MPFI. Then, we used the following formula to calculate fluorescence intensity: $FL\ Intensity\ (a.u.)\ =\ MPFI(on-tissue) - MPFI(off-tissue)$. The uncoated tissue halves served as internal controls and had the same values as the entirely uncoated control (none).

For calculating fold enhancement for tissue sections, the following formula was used: $Fold\ Enhancement = \frac{MPFI(on-tissue\ matrix)/MPFI(off-tissue\ matrix)}{MPFI(on-tissue\ none)/MPFI(off-tissue\ none)}$.

MALDI Mass Spectrometry Imaging – Additional Details

For timsTOF measurements of mouse kidney sections in **Fig. S6** in positive ion mode, prior to data acquisition, height adjustment (target profile generation), laser focus tuning, and mass calibration were conducted. Electrospray ionization (ESI) of Agilent ESI-L Tune Mix was used to perform mass calibration, followed by calibration with red phosphorus to achieve a mass error <1 PPM. MALDI parameters in qTOF mode were optimized to maximize intensity by tuning ion optics, laser intensity,

and laser focus. Additional experimental values include: MALDI plate offset of 50 V, deflection 1 delta of 70 V, funnel 1 RF of 350 Vpp, funnel 2 RF 350 Vpp, multipole RF of 350 Vpp, and a collision cell energy of 10 eV, a collision RF of 2500 Vpp, quadrupole ion energy 5 eV with low mass of m/z 300 Da, focus pre TOF transfer time of 80 μs, and a prepulse storage time of 10 μs, and both high sensitivity detection and focus mode turned off. All images were acquired for m/z 300-1000 Da in positive ion mode at 50 μm raster width with 200 shots per pixel using the single laser with beam scan range of 46 μm resulting in 50 μm field size and 10 kHz frequency.

For timsTOF measurements of mouse kidney sections in **Fig. S6** in negative ion mode, prior to data acquisition, height adjustment (target profile generation), laser focus tuning, and mass calibration were conducted. ESI of Agilent ESI-L Tune Mix was used to perform mass calibration, followed by calibration with red phosphorus to achieve a mass error <1 PPM. MALDI parameters in qTOF mode were optimized to maximize intensity by tuning ion optics, laser intensity, and laser focus. Additional experimental values include: MALDI plate offset of 50 V, deflection 1 delta of -70 V, funnel 1 RF of 350 Vpp, funnel 2 RF 350 Vpp, multipole RF of 350 Vpp, and a collision cell energy of 10 eV, a collision RF of 2500 Vpp, quadrupole ion energy 5 eV with low mass of m/z 300 Da, focus pre TOF transfer time of 110 μs, and a prepulse storage time of 5 μs, and both high sensitivity detection and focus mode turned off. All images were acquired for m/z 500-1500 Da in negative ion mode at 50 μm raster width with 200 shots per pixel using the single laser with beam scan range of 46 μm resulting in 50 μm field size and 10 kHz frequency.

On-tissue tandem MS of m/z^+ 478.33 Da and m/z^+ 504.04 Da was performed on timsTOF fleX with nitrogen as a collision gas, acquiring a sum of 5 spectra using 200 shots per pixel for m/z 50-1000 Da at 10 kHz frequency. The isolation window was ±1 Da. For m/z^- 906.63 Da with nH matrix, the laser power was set to 80% and the collision energy was 85 eV. For m/z^- 621.30 Da with 9AA matrix, the laser power was set to 80% and the collision energy was 20 eV. For m/z^+ 785.45 Da with CHCA matrix, the laser power was set to 86% and the collision energy was 45 eV.

MALDI MSI Data Processing and Analysis – Additional Details

For MALDI imaging experiments on timsTOF fleX shown in **Fig. S6** for mouse kidney sections, MALDI MSI data were imported into SCiLS Lab (v 2023b, Bruker Daltonics) and segmentation analysis was conducted on TIC normalized data through the software's segmentation pipeline, which conducts peak selection and peak alignment and smoothing prior to segmentation using the bisecting k-means algorithm in combination with correlation distance. Segmentation maps were generated and exported from SCiLS Lab. Individual representative m/z features from each segment were identified from the segmentation analysis and the corresponding results were then visualized in flexImaging. Tandem MS spectra were exported from dataAnalysis (version 5.3.236, Bruker Daltonics).

Single-crystal X-ray Crystallography – additional details

A mixture of 1:1.25 CHCA and rhodamine B dissolved in 50% toluene was prepared in a single vial. Subsequently, this mixture was allowed to evaporate at room temperature. After one week, several red crystals were obtained in block form. All reflection intensities were measured at 110.00(10) K using a Rigaku XtaLAB Synergy R (Rigaku) equipped with a rotating-anode X-ray source and HyPix-6000HE detector with Cu *K*α radiation (λ = 1.54178 Å) using CrysAlisPro (Version CrysAlisPro 1.171.42.49, Rigaku OD, 2022). The temperature of the data collection was controlled using a Cryostream 1000

system (Oxford Cryosystems, Oxford, United Kingdom). CrysAlisPro was also used to refine the cell dimensions and for data reduction. The structure was solved with the SHELXT-2018/2 software (Sheldrick, 2018) and was refined on F^2 with SHELXL-2019/3 (Sheldrick, 2018). Analytical numeric absorption correction using a multifaceted crystal was applied using CrysAlisPro. The H atoms were placed at calculated positions (unless otherwise specified) using the instructions AFIX 23, AFIX 43 or AFIX 137 with isotropic displacement parameters having values 1.2 or 1.5 U_{eq} of the attached C atoms. The H atom attached to O3/O4 and O6 were found from difference Fourier maps. The coordinates for H6O (attached to O6) were refined pseudo-freely using the DFIX instruction to keep the O–H distance within an acceptable range. The structure is partly disordered. The moiety C14–C15 is disordered over two orientations, and the occupancy factor of the major component of the disorder refines to 0.743(6).

Response to Alert Level A (from checkCIF):
PLAT355_ALERT_3_A Long O-H (X0.82,N0.98A) O4 - H4O . 1.16 Ang.
The H atom H4O lies at a position somewhat intermediate between O3 and O4 (this is observed from the contoured difference Fourier map drawn in the plane defined by O3, O4 and H4O, see Figure S11) as there is a strong hydrogen bond interaction found between the carboxylic acid – carboxylate pair (O3…O4: 2.4610(16) Å). The coordinates for H4O were refined freely in the final refinement. Most likely, CHCA and Rhodamine B can act both as carboxylic acid and carboxylate without any preference in the crystal. Hence, the unusually long O–H distance is an experimental artifact resulting from a space average over all possible positions of the O–H in the crystal.

Specified hydrogen bonds (with esds except fixed and riding H)

D-H	H...A	D...A	<(DHA)	
1.15(3)	1.31(3)	2.4610(16)	174(2)	O4-H4O...O3
0.95(2)	1.72(2)	2.6318(18)	160(2)	O6-H6O...O2_$3

Crystallographic Data of CHCA_RhodamineB

	**CHCA_RhodamineB**
Crystal data	
Chemical formula	$C_{28}H_{30}N_2O_3 \cdot C_{10}H_7NO_3$
M_r	631.70
Crystal system, space group	Triclinic, *P*-1
Temperature (K)	110
a, *b*, *c* (Å)	11.5312 (3), 12.0186 (3), 12.6873 (4)
α, β, γ (°)	109.995 (2), 91.245 (2), 93.597 (2)
V (Å^3)	1647.33 (8)
Z	2
Radiation type	Cu *K*α
μ (mm^{-1})	0.70

Crystal size (mm)	0.16 × 0.07 × 0.03
Data collection	
Diffractometer	XtaLAB Synergy R, HyPix
Absorption correction	Analytical<br>*CrysAlis PRO* 1.171.42.80a (Rigaku Oxford Diffraction, 2023) Analytical numeric absorption correction using a multifaceted crystal model based on expressions derived by R.C. Clark & J.S. Reid. (Clark, R. C. & Reid, J. S. (1995). Acta Cryst. A51, 887-897) Empirical absorption correction using spherical harmonics, implemented in SCALE3 ABSPACK scaling algorithm.
T_{min}, T_{max}	0.934, 0.987
No. of measured, independent and observed [$I > 2\sigma(I)$] reflections	32312, 6460, 5364
R_{int}	0.029
$(\sin \theta/\lambda)_{max}$ (Å^{-1})	0.617
Refinement	
$R[F^2 > 2\sigma(F^2)]$, $wR(F^2)$, S	0.047, 0.144, 1.05
No. of reflections	6460
No. of parameters	454
No. of restraints	33
H-atom treatment	H atoms treated by a mixture of independent and constrained refinement
$\Delta\rho_{max}$, $\Delta\rho_{min}$ (e Å^{-3})	0.36, -0.25

Computer programs: *CrysAlis PRO* 1.171.42.49 (Rigaku OD, 2022), *SHELXT2018*/2 (Sheldrick, 2018), *SHELXL2019*/3 (Sheldrick, 2018), *SHELXTL* v6.10 (Sheldrick, 2008).

References: Sheldrick, G. M. (2015). Acta Cryst. C71, 3-8.

Supporting Tables

Table S1. Assignment of MALDI-MSI signals of endogenous lipids in mouse brain tissue using various matrices in negative ion mode.

Matrix						References
CHCA		9AA		nH		
m/z value	Assignment [M-H]$^-$	*m/z* value	Assignment [M-H]$^-$	*m/z* value	Assignment [M-H]$^-$	
788.8	PS (36:1)	806.5	PS (18:0)	747.8	PA (18:0/22:6)	Jackson et al., 2005; Jackson et al., 2007; Cerruti et al., 2012; Yang et al., 2013; Liu et al., 2014; Liu et al., 2018
806.5	PS (18:0)	821.8	PG (40:6)	762.8	PE (16:0/22:6)	
862.8	ST (22:0)	822.8	ST (18:0)(OH)	774.8	PE (P-18:0/22:6)	
878.8	ST (22:0)(OH)	834.8	PS (18:0/22:6)	786.8	PC (36:1)	
885.8	PI (38:4)	857.8	PI (16:0/20:4)	806.5	PS (18:0)	
888.8	ST (24:1)	862.8	ST (22:0)	834.8	PS (18:0/22:6)	
889.8	PI (40:6)	878.8	ST (22:0)(OH)	857.8	PI (16:0/20:4)	
890.8	ST (24:0)	879	PI (37:0)	862.8	ST (22:0)	
904.8	ST (24:1)(OH)	885.8	PI (38:4)	878.8	ST (22:0)(OH)	
906.8	SHexCer (42:1);O3	888.8	ST (24:1)	879	PI (37:0)	
		889.8	PI (40:6)	885.8	PI (38:4)	
		890.8	ST (24:0)	888.8	ST (24:1)	
		904.8	ST (24:1)(OH)	889.8	PI (40:6)	
		906.8	SHexCer (42:1);O3	890.8	ST (24:0)	
		908	PI (40:6)	904.8	ST (24:1)(OH)	
				906.8	SHexCer (42:1);O3	

1. Cerruti, C.D., Benabdellah, F., Laprévote, O., Touboul, D., Brunelle, A. . MALDI imaging and structural analysis of rat brain lipid negative ions with 9-aminoacridine matrix. *Anal Chem* **84**, 2164-2171 (2012).

2. Jackson, S.N., Wang, H.Y., Woods, A.S. In situ structural characterization of glycerophospholipids and sulfatides in brain tissue using MALDI-MS/MS. *J Am Soc Mass Spectrom* **18**, 17-26 (2007).

3. Jackson, S.N., Wang, H.Y., Woods, A.S. Direct profiling of lipid distribution in brain tissue using MALDI-TOFMS. *Anal Chem* **77**, 4523-4527 (2005).

4. Liu, H., Zhou, Y., Wang, J., Xiong, C., Xue, J., Zhan, L., Nie, Z. N-Phenyl-2-naphthylamine as a novel MALDI matrix for analysis and in situ imaging of small molecules. *Anal Chem* **90**, 729-736 (2018)

5. Liu, H., Chen, R., Wang, J., Chen, S., Xiong, C., Wang, J., Hou, J., He, Q., Zhang, N., Nie, Z., Mao, L. 1,5-Diaminonaphthalene hydrochloride assisted laser desorption/ionization mass spectrometry imaging of small molecules in tissues following focal cerebral ischemia. *Anal Chem* **86**, 10114-10121 (2014).

6. Yang, J., Caprioli, R.M. Matrix precoated targets for direct lipid analysis and imaging of tissue. *Anal Chem* **85**, 2907-2912 (2013).

Table S2. Effects of fluorescence slide scanning on MALDI imaging of lipid signals in mouse brain tissue sections in positive and negative ion modes.

m/z **value (brain region)**		**Matrix**								
		**CHCA**			**9AA**			**nH**		
		**Green channel**	**Red channel**	**All channels**	**Green channel**	**Red channel**	**All channels**	**Green channel**	**Red channel**	**All channels**
**$[M+H]^+$ 478 (hippocampus)**	**No FL control**	236.5	449.4	220.3						
	**FL**	247.2 (104.5%)	446.2 (99.3%)	105.5 (47.9%)						
**$[M+H]^+$ 478 (hemisphere)**	**No FL control**	200.6	340.8	143.3						
	**FL**	189.1 (94.3%)	332.9 (97.7%)	93 (64.9%)						
**$[M+Na]^+$ 504 (hippocampus)**	**No FL control**	77.4	166.8	190.7						
	**FL**	82.4 (106.5%)	168.1 (100.8%)	131 (68.7%)						
**$[M+Na]^+$ 504 (hemisphere)**	**No FL control**	88.5	168.3	146.2						
	**FL**	81.7 (92.3%)	164.9 (98%)	120.8 (82.6%)						
**$[M-H]^-$ 885 (hippocampus)**	**No FL control**				7.2	6	32.4	241	129.1	170.8
	**FL**				7.1 (98.6%)	6.2 (103.3%)	22.6 (69.8%)	228 (94.6%)	129.7 (100.5%)	150 (87.9%)
**$[M-H]^-$ 885 (hemisphere)**	**No FL control**				9.9	20.1	35.2	151.9	190.8	183.1
	**FL**				9.2 (92,9%)	22.8 (113.7%)	23.8 (67.6%)	156.5 (103%)	199.9 (104.8%)	170 (92.8%)
**$[M-H]^-$ 888 (hippocampus)**	**No FL control**				16.9	27.7	31.9	82	41.5	112.6
	**FL**				16.8 (99.4%)	28.8 (103.9%)	16.9 (54.2%)	87 (106.1%)	46.1 (111.1%)	87.6 (77.8%)
**$[M-H]^-$ 888 (hemisphere)**	**No FL control**				36.6	200.2	191.9	207	149.3	365.6
	**FL**				32.5 (88.8%)	169.9 (84.9%)	124.3 (64.8%)	196 (94.7%)	138.3 (92.6%)	336 (91.9%)

1. All values represent total ion count (TIC)-nomalized intensities.

2. The exposure time for the green channel alone was set to 79 ms and for the red channel alone to 78 ms, The exposure time for the acquisition of consecutive channels (blue, green, and red) was set to 150 ms per channel.

3. The calculation of percentage (%) in the table represents the ratio of MS intensity between fluorescence-exposed tissue (FL) and non-fluorescence-exposed tissue (No FL Control): [(FL/No FL control) x 100]

Supporting Figures

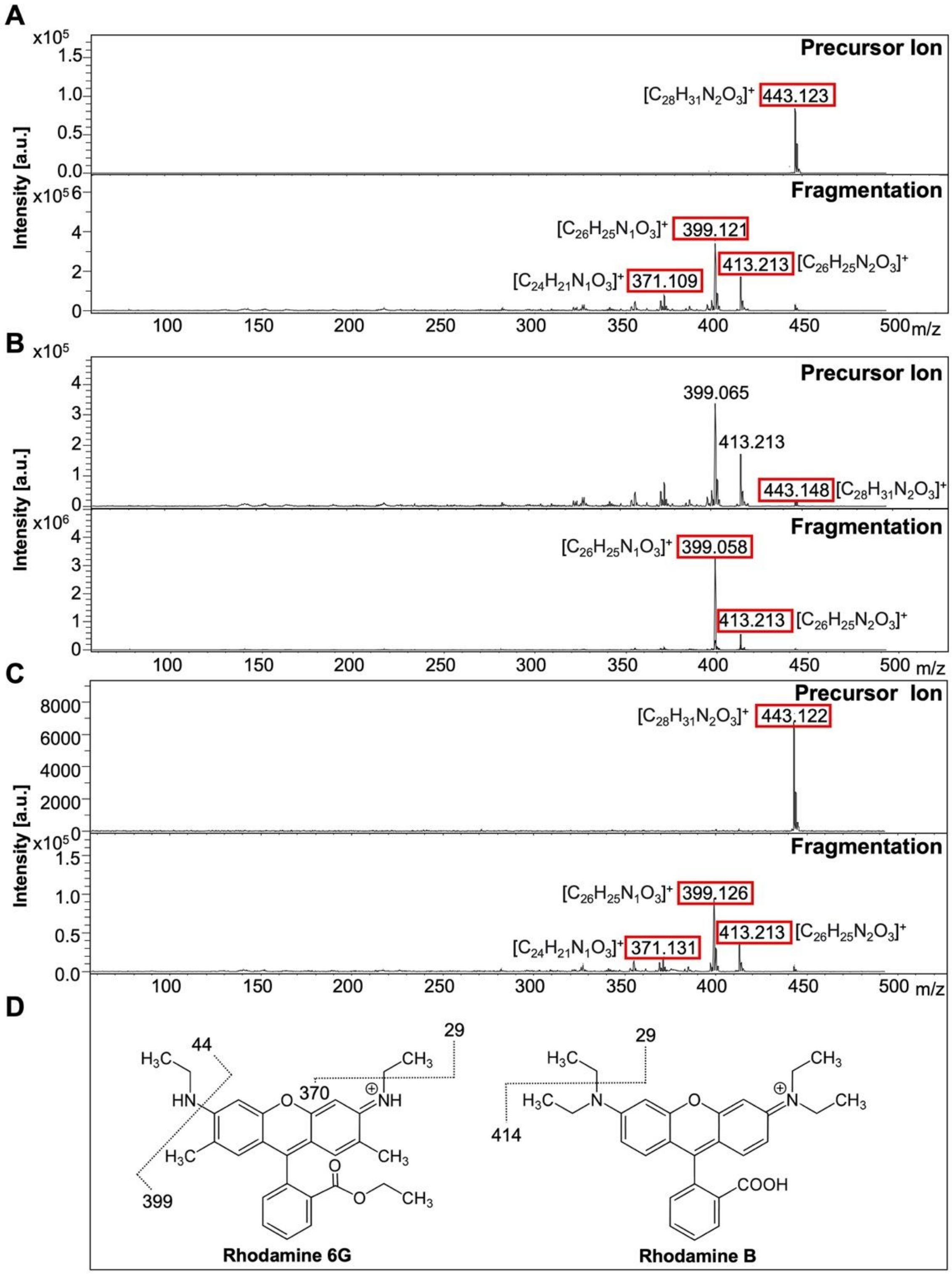

Figure S1. Collision induced dissociation (CID) tandem MS spectra of Rhodamine B and pink Sharpie® permanent marker drawings in positive ion mode. (**A**) Precursor ion at m/z^+ 443.122 Da (top) of pure Rhodamine B and resulting fragment ions (bottom). (**B**) Precursor ion at m/z^+ 443.122 Da (top) and fragment ions (bottom) of pink Sharpie® permanent marker drawings without matrix. Significant in source fragmentation is observed for the precursor ion. (**C**) Precursor ion at m/z^+ 443.122 Da (top) and fragment ions (bottom) of pink Sharpie® permanent marker drawings coated with the MALDI matrix DAN. (**D**) Chemical structures of Rhodamine 6G and B are shown, including characteristic fragmentation giving rise to diagnostic ions as boxed in red in S1A-C.

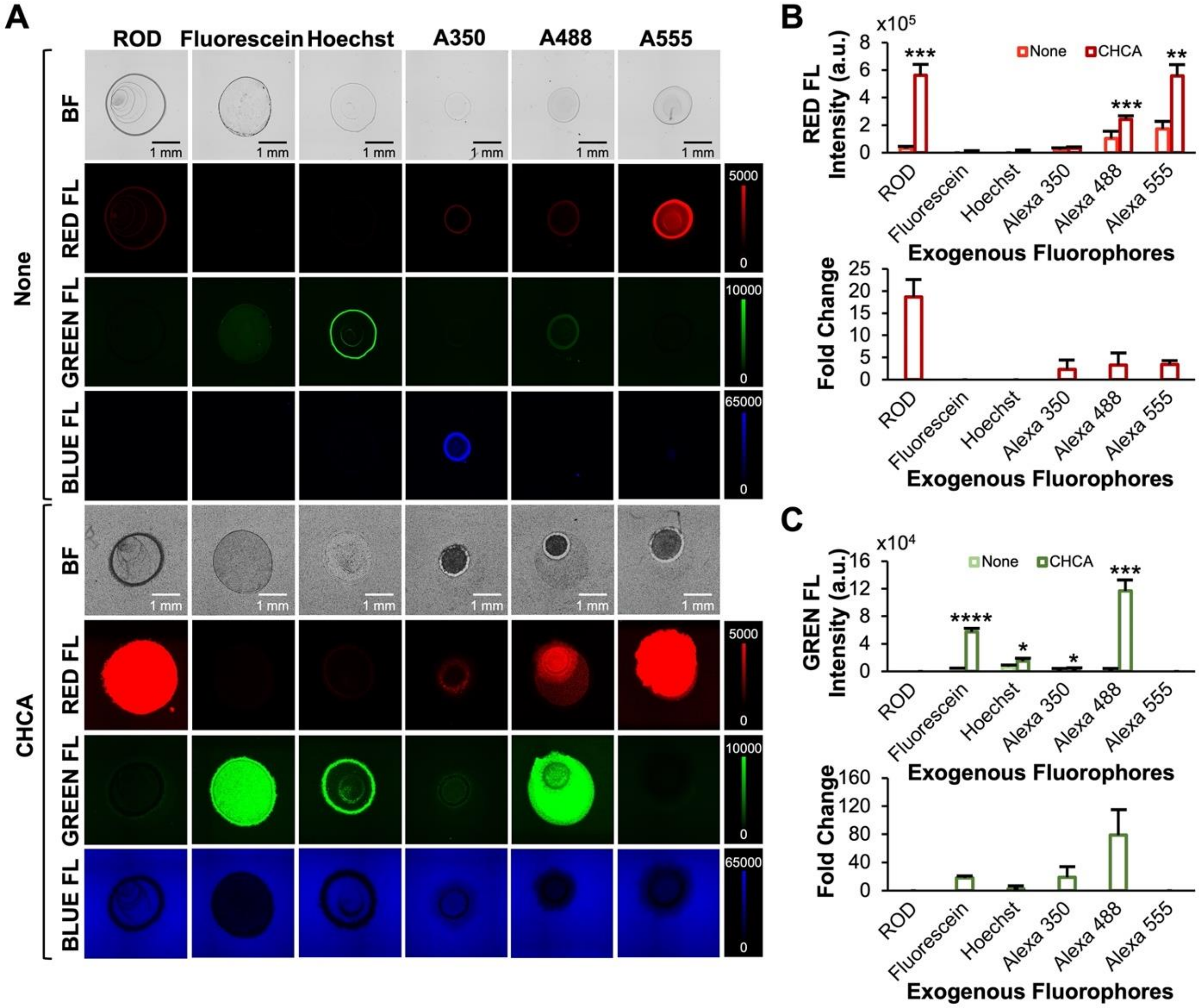

Figure S2. Fluorescence intensity and fold change in fluorescence enhancement of exogenous fluorophores coated with CHCA matrix. (**A**) Six common exogenous fluorophores including Rhodamine B, Fluorescein, Hoechst, Alexa Fluor 350 (A350), Alexa Fluor 488 (A488), and Alexa Fluor 555 (A555) were spotted onto slides at equal concentrations, dried, and sprayed with CHCA matrix at 1.6 µg/mm^2 density. Red, green, and blue epifluorescence images were acquired from uncoated fluorophores (none, top) and CHCA-coated fluorophores (bottom). (**B**) Quantification of red fluorescence intensities and fold change from CHCA-coated exogenous fluorophores compared to respective uncoated controls, n=3. (**C**) Quantification of green fluorescence intensities and fold change from CHCA-coated exogenous fluorophores compared to respective uncoated controls, n=3. Blue fluorescence intensities were not quantified because of high innate blue fluorescence of CHCA matrix causing high background signal. All quantitative data are shown as mean values ± standard error of three independent experiments. * p<0.05, ** p<0.01, *** p<0.001. Abbreviations: BF, brightfield; FL, fluorescence; ROD, Rhodamine B.

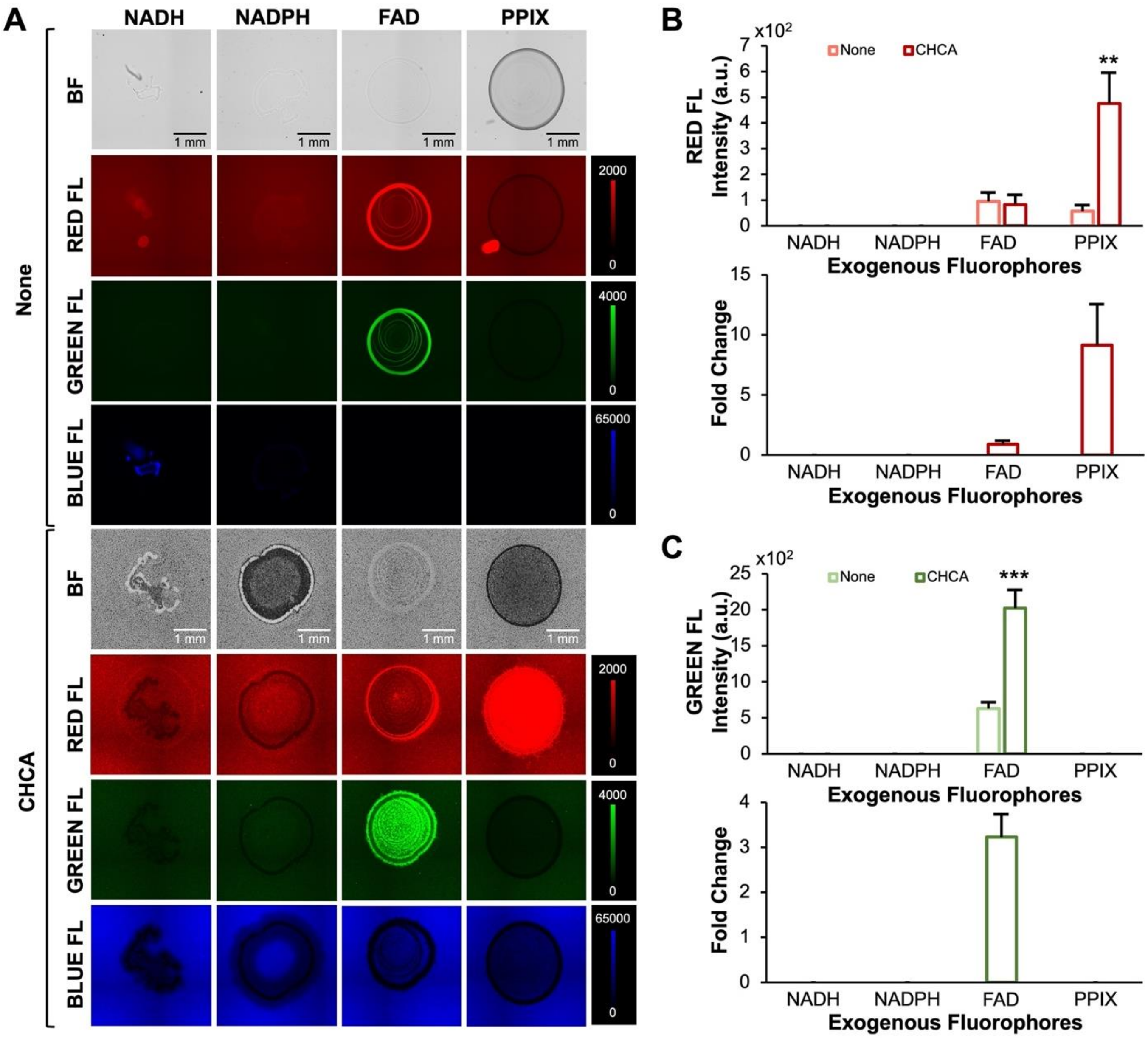

Figure S3. Fluorescence intensity and fold change in fluorescence enhancement of endogenous fluorophores coated with CHCA matrix. (**A**) Four common endogenous fluorophores including reduced nicotinamide adenine dinucleotide (NADH), reduced nicotinamide adenine dinucleotide phosphate (NADPH), flavin adenine dinucleotide (FAD), and protoporphyrin IX (PPIX) were spotted onto slides at equal concentrations, dried, and sprayed with CHCA matrix at 1.6 µg/mm^2 density. Red, green, and blue epifluorescence images were acquired from uncoated fluorophores (none, top) and CHCA-coated fluorophores (bottom). (**B**) Quantification of red fluorescence intensities and fold change from CHCA-coated endogenous fluorophores compared to respective uncoated controls, n=3. (**C**) Quantification of green fluorescence intensities and fold change from CHCA-coated endogenous fluorophores compared to respective uncoated controls, n=3. Blue fluorescence intensities were not quantified because of high innate blue fluorescence of CHCA matrix causing high background signal. All quantitative data are shown as mean values ± standard error of three independent experiments. ** $p<0.01$, *** $p<0.001$. Abbreviations: BF, brightfield; FL, fluorescence.

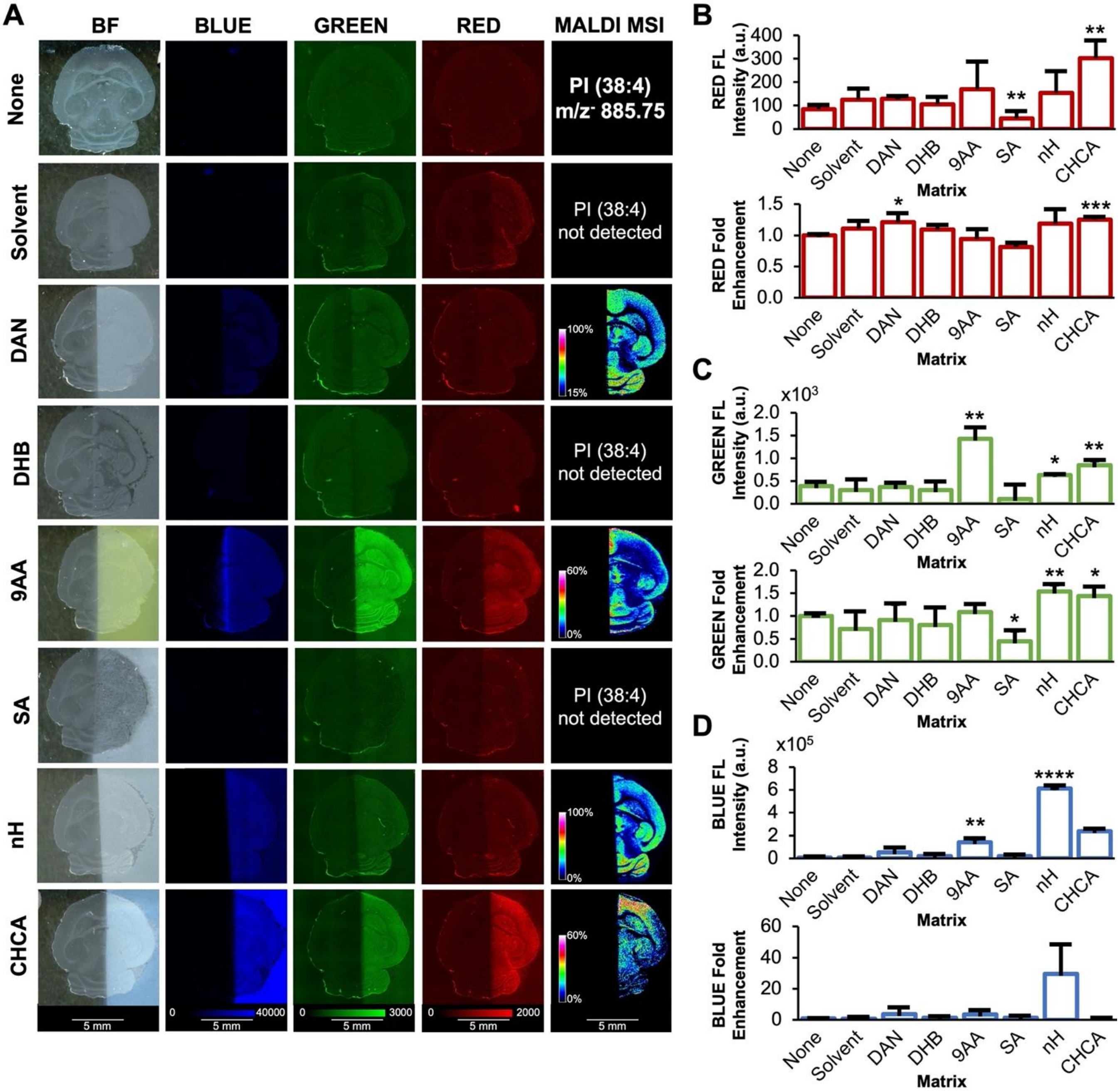

Figure S4. MALDI matrix coating of mouse brain tissue sections increases tissue autofluorescence intensity. (**A**) Imaging results of transverse (axial) mouse brain tissue sections showing, in columns from left to right, brightfield, green fluorescence, red fluorescence, and MALDI imaging in negative ion mode displaying m/z^- 885.75 Da, which was identified by tandem MS as phosphatidylinositol (PI) (38:4) ($[M\text{-}H]^-$, see **Fig. S5** for tandem MS data). Rows show uncoated controls and coating with various MALDI matrices at 1.6 µg/mm^2 density. Fluorescence and MALDI images are shown on the same intensity scale per column. Quantification of (**B**) red, (**C**) green, and (**D**) blue fluorescence intensities from (A), n=3. All quantitative data are shown as mean values ± standard error of three independent experiments. * p<0.05, ** p<0.01, *** p<0.001. Abbreviations: BF, brightfield; FL, fluorescence.

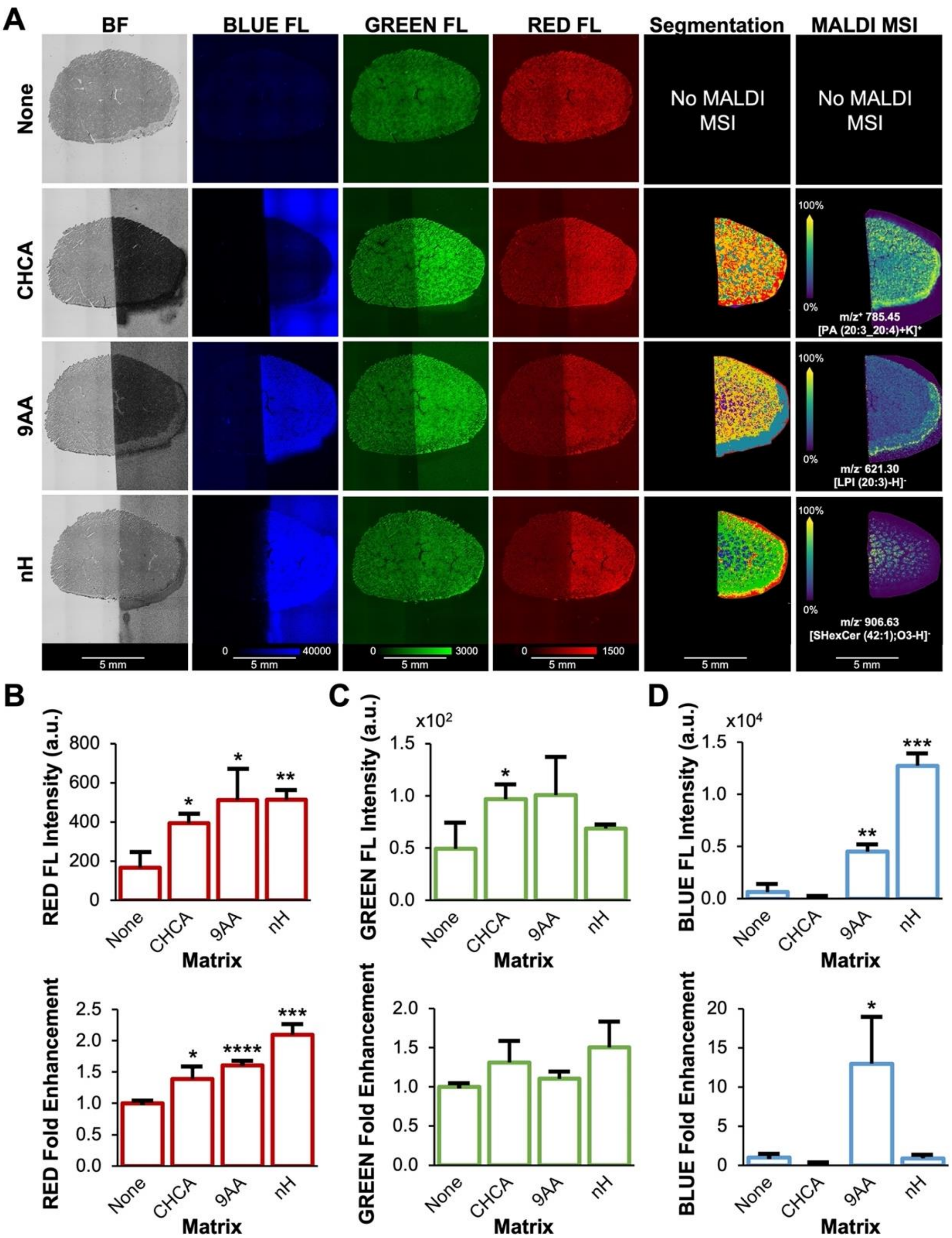

Figure S5. MALDI matrix coating of mouse kidney tissue sections increases tissue autofluorescence intensity. (**A**) Imaging results of coronal mouse kidney tissue sections showing, in columns from left to right, brightfield, blue fluorescence, green fluorescence, red fluorescence, segmentation analysis from MALDI imaging, MALDI imaging displaying m/z^+ 785.45 Da for CHCA matrix identified by on-tissue tandem MS as phosphatidic acid (PA) (20:3_20:4) $[M+K]^+$, (see **Fig. S7** for tandem MS data), m/z^- 621.30 Da for 9AA matrix identified by on-tissue tandem MS as lyso-phosphatidylinositol (LPI) (20:3) $[M-H]^-$ (see **Fig. S8** for tandem MS data), and m/z^- 906.63 Da for nH matrix identified by on-tissue tandem MS as sulfatide (SHexCer) (42:1);O3 $[M-H]^-$ (see **Fig. S9** for tandem MS data). Rows show uncoated control and coating with various MALDI matrices at 1.6 µg/mm^2 density. Fluorescence and MALDI images are shown on the same intensity scale per column. Quantification of (**B**) blue, (**C**) green, and (**D**) red fluorescence intensities from (A), n=3. All quantitative data are shown as mean values ± standard error of three independent experiments. * p<0.05, ** p<0.01, *** p<0.001, **** p<0.0001. Abbreviations: BF, brightfield; FL, fluorescence.

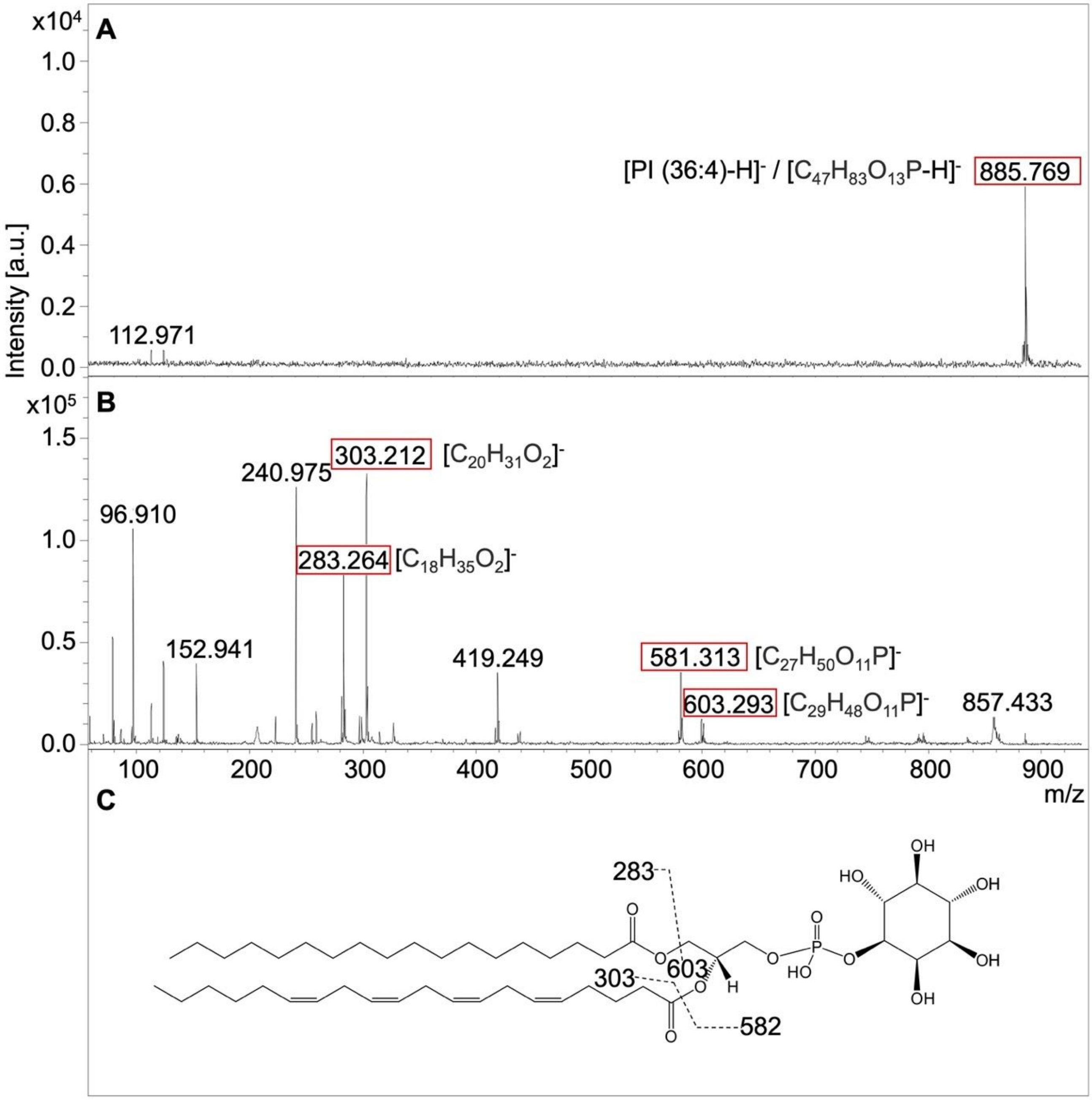

Figure S6. Negative ion mode tandem MS spectra of m/z$^-$ 885.8 Da identified as PI (38:4), [M-H]$^-$. (**A**) Precursor ion at m/z$^-$ 885.8 Da, and (**B**) fragmentation of m/z$^-$ 885.8 Da. Chemical structures of characteristic fragments (boxed in red) are shown.

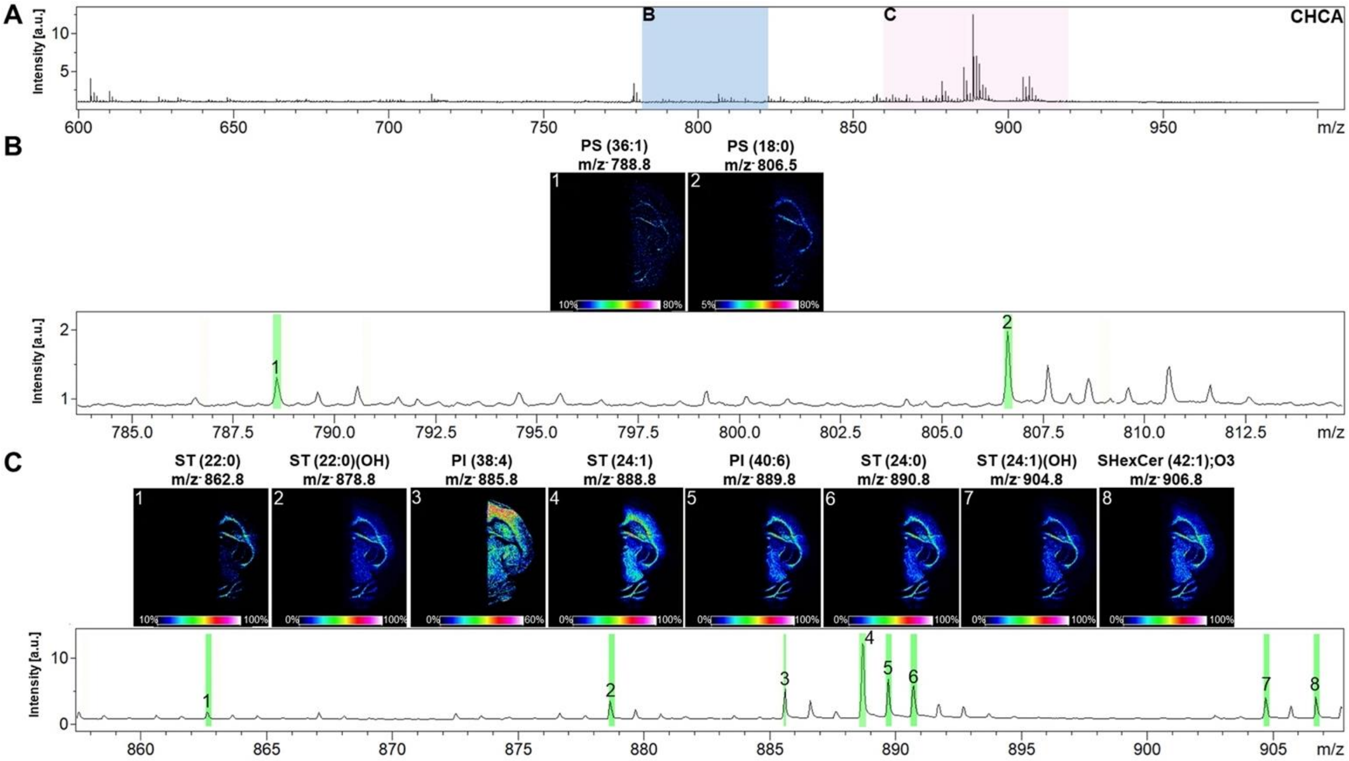

Figure S7. Average positive ion mode spectra of MALDI imaging data and corresponding m/z images from the CHCA-coated half of the mouse brain tissue sections shown in Figure 3. (**A**) Average MALDI imaging spectrum for the CHCA-coated half of the mouse brain tissue sections shown in Figure 3. Highlighted regions in (**B**) blue and (**C**) red are expanded from (A) as indicated, and numbered peaks are shown as corresponding m/z images.

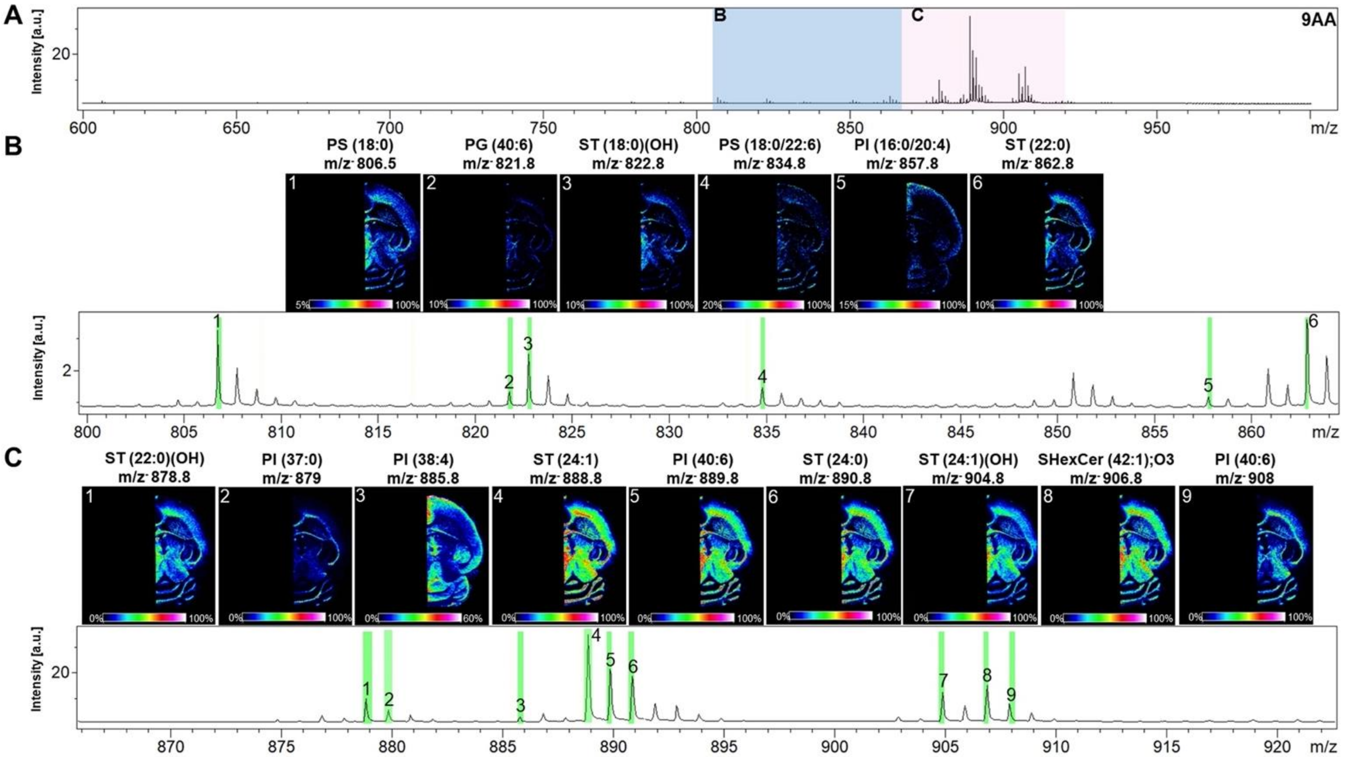

Figure S8. Average negative ion mode spectra of MALDI imaging data and corresponding m/z images from the 9AA-coated half of the mouse brain tissue sections shown in Figure 3. (**A**) Average MALDI imaging spectrum for the 9AA-coated half of the mouse brain tissue sections shown in Figure 3. Highlighted regions in (**B**) blue and (**C**) red are expanded from (A) as indicated, and numbered peaks are shown as corresponding m/z images.

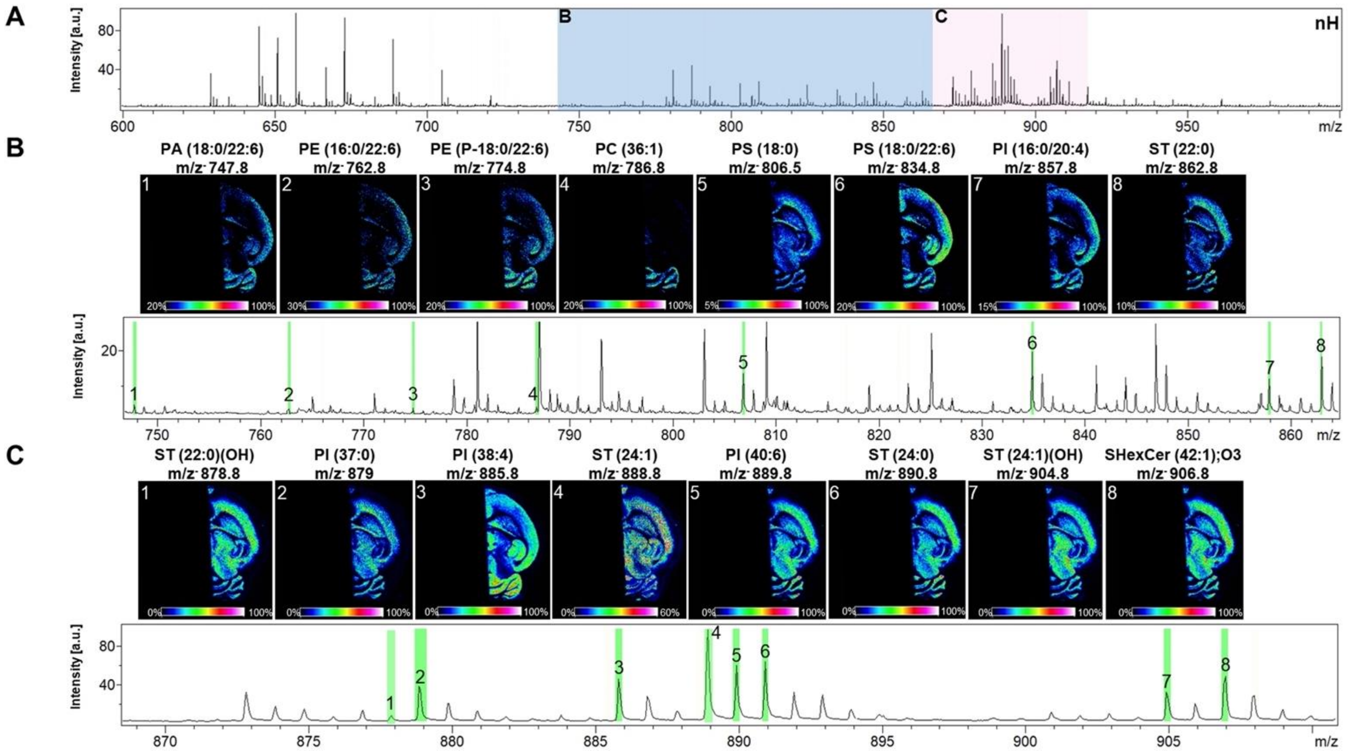

Figure S9. Average negative ion mode spectra of MALDI imaging data and corresponding m/z images from the nH-coated half of the mouse brain tissue sections shown in Figure 3. (**A**) Average MALDI imaging spectrum for the nH-coated half of the mouse brain tissue sections shown in Figure 3. Highlighted regions in (**B**) blue and (**C**) red are expanded from (A) as indicated, and numbered peaks are shown as corresponding m/z images.

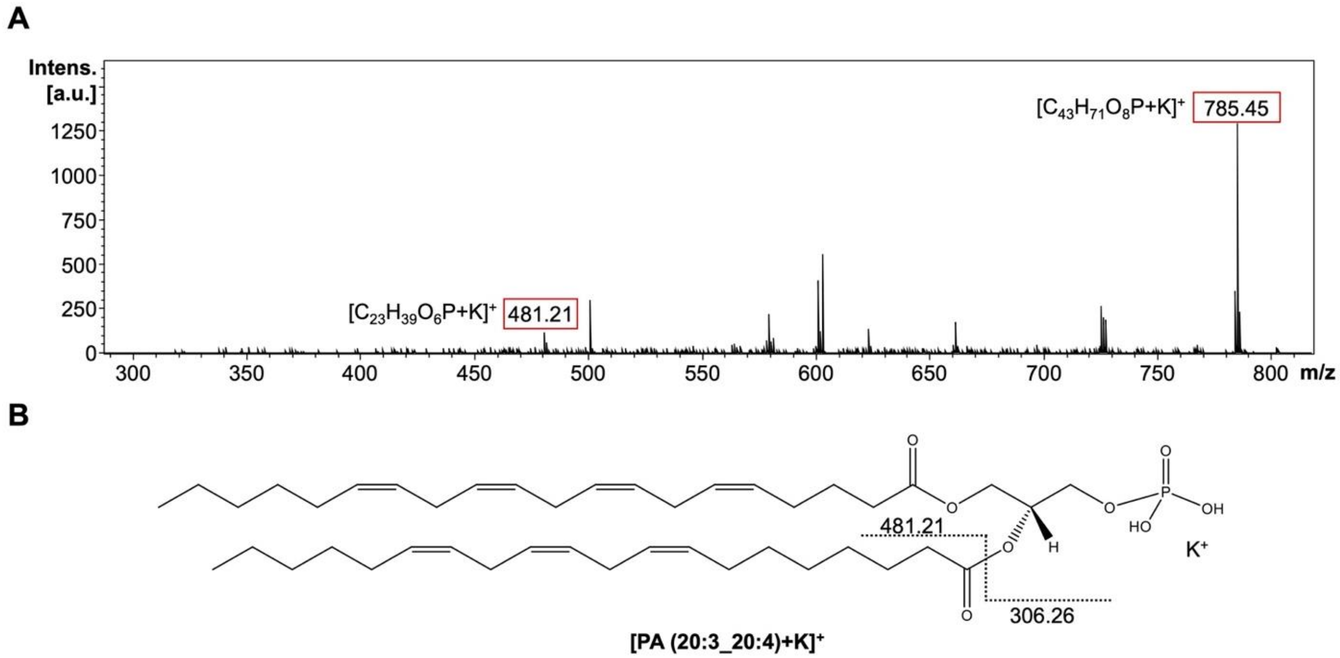

Figure S10. Positive ion mode tandem MS spectra of m/z+ 785.45 Da identified as PA (20:3_20:4), [M+K]+.
(**A**) Precursor ion and fragmentation of m/z+ 785.45 Da. (**B**) Chemical structure and fragmentation are shown based on characteristic fragments (boxed in red).

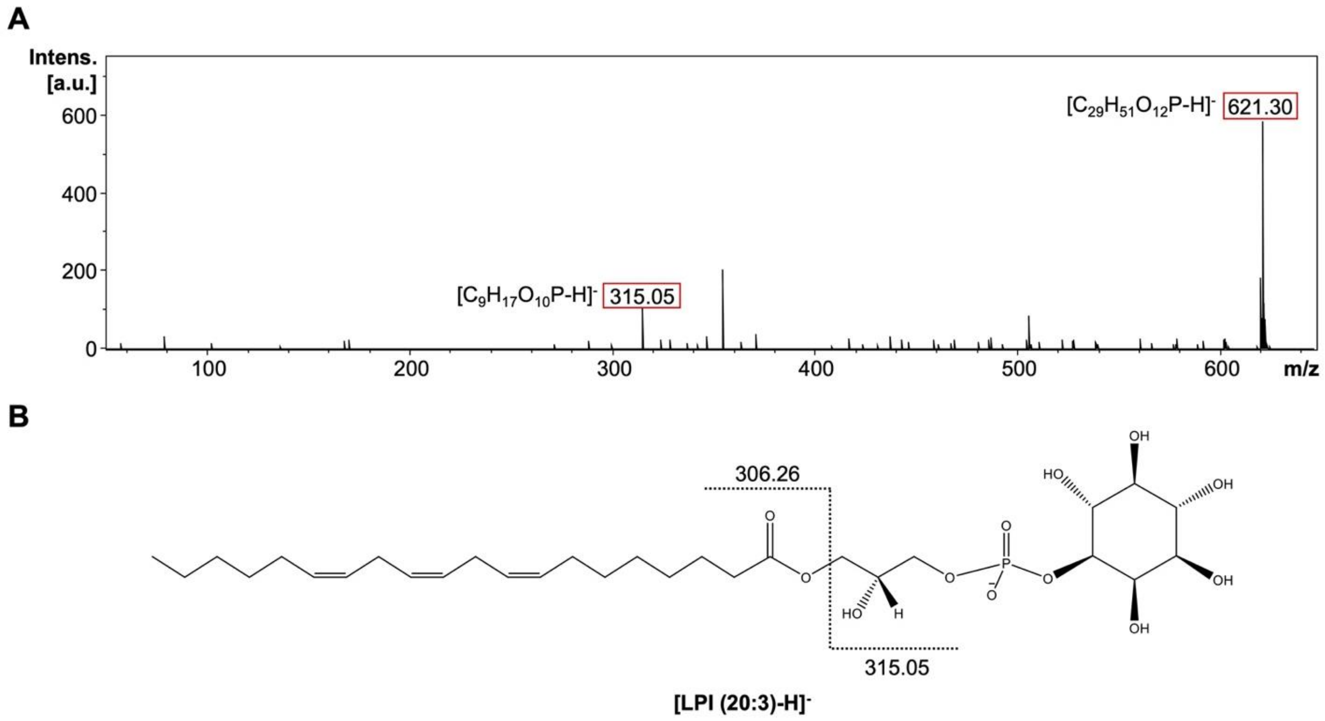

Figure S11. Negative ion mode tandem MS spectra of m/z⁻ 621.30 Da identified as LPI (20:3), [M-H]⁻.
(**A**) Precursor ion and fragmentation of m/z⁻ 621.30 Da. (**B**) Chemical structure and fragmentation are shown based on characteristic fragments (boxed in red).

A

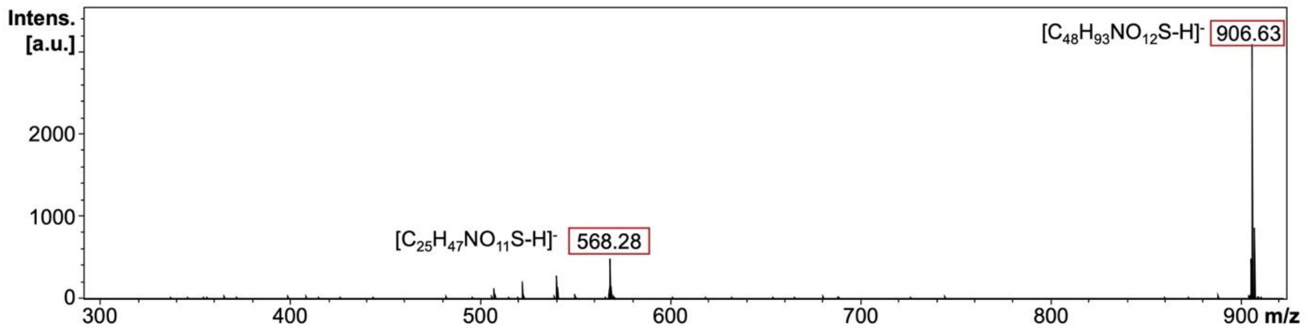

B

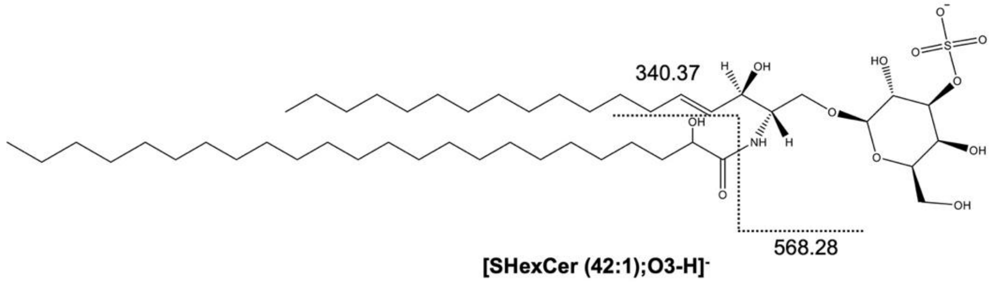

Figure S12. Negative ion mode tandem MS spectra of m/z⁻ 906.63 Da identified as SHexCer (42:1);O3, [M-H]⁻.
(**A**) Precursor ion and fragmentation of m/z⁻ 906.63 Da. (**B**) Chemical structure and fragmentation are shown based on characteristic fragments (boxed in red).

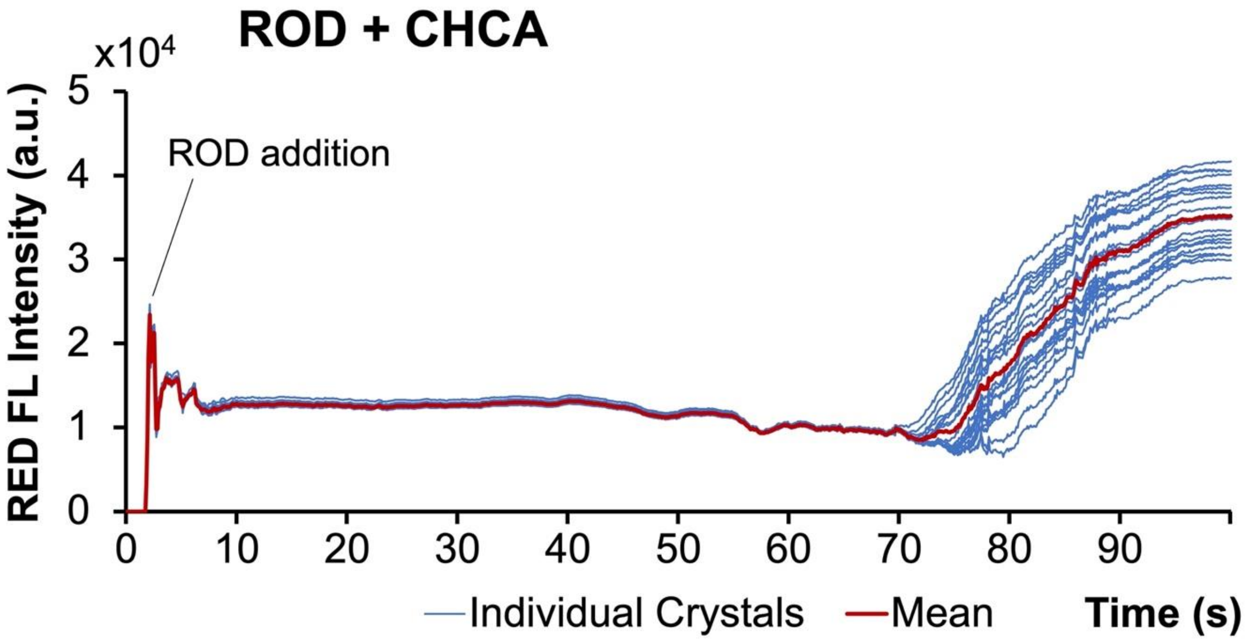

Figure S13. Co-crystallization of fluorophores with matrices – full length time course for ROD+CHCA. Full length time course of fluorescence intensity quantification of the 20 largest crystals for (co-)crystallization of ROD+CHCA. Blue lines represent individual crystals' fluorescence intensities, red lines are mean crystal fluorescence intensities. Time point of adding ROD to matrix solution on microscope slide is pointed out. This data is also available as **video V4**.

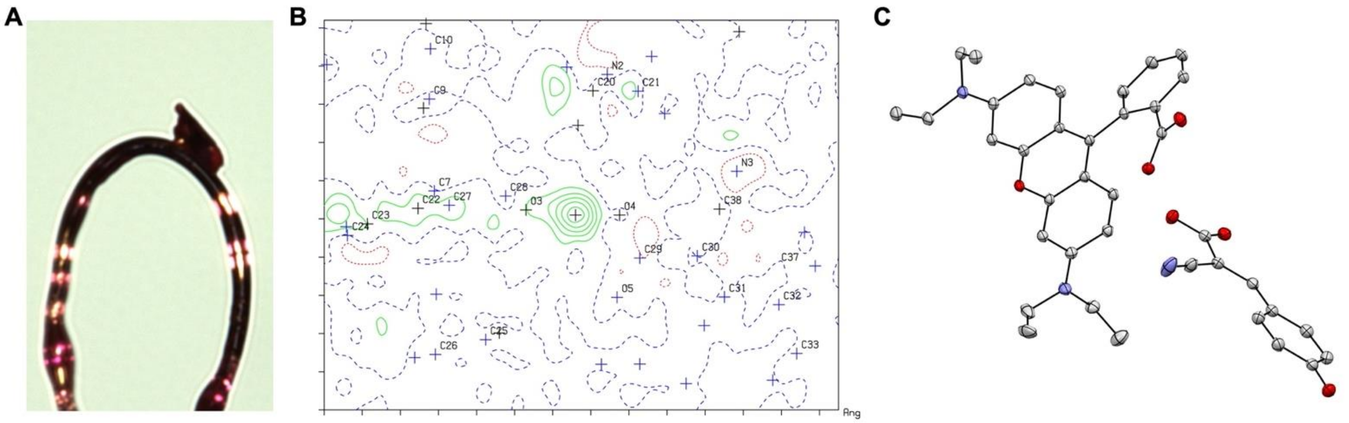

Figure S14. Single-crystal X-ray crystallography of ROD+CHCA. (**A**) Photograph of ROD+CHCA co-crystal mounted on a loop used for single crystal X-ray crystallography measurements. (**B**) Contoured difference Fourier map drawn in the plane defined by the atoms O3, O4 and H4O. The residual electron density peak (i.e., one H atom) is located at an intermediate position between O3 and O4, which suggests that one H atom is shared by O3 and O4 in the crystal. Most likely, both CHCA and ROD can be found as pairs of carboxylic acid – carboxylate and vice versa. (**C**) Displacement ellipsoid plot (50% probability level) of the asymmetric unit of ROD+CHCA at 110.00(10) K. H atoms and disorder were removed for clarity.

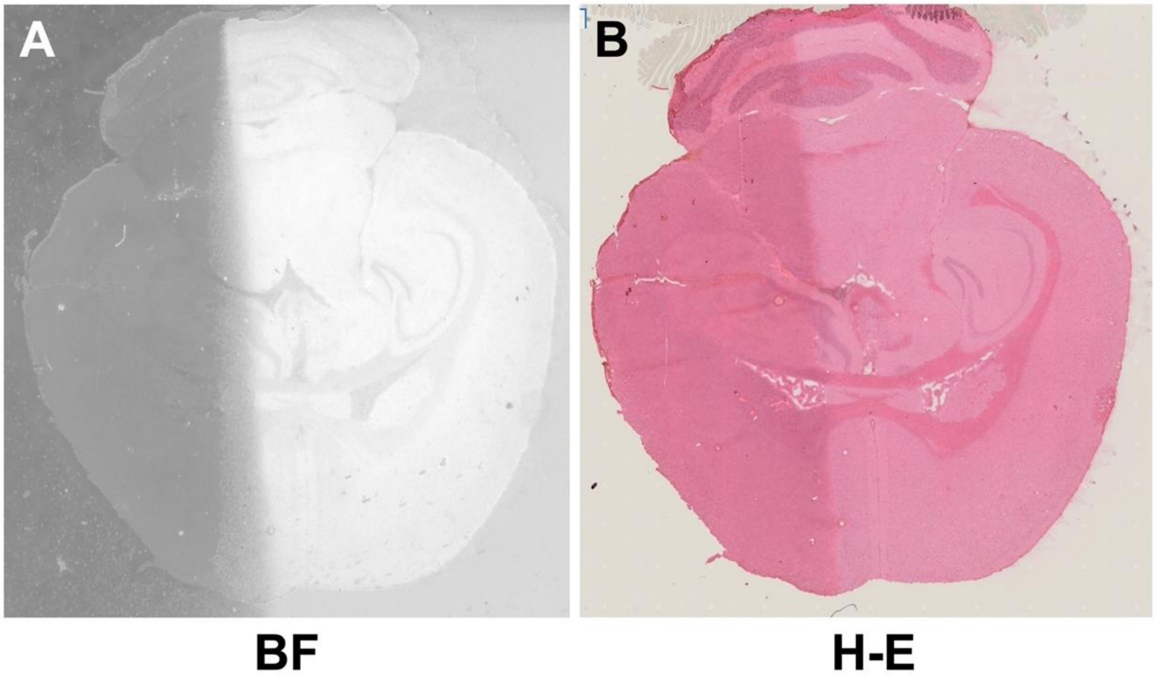

Figure S15. Brightfield and H-E-stained images of transverse (axial) half-coated brain section shown in Fig. 5. (**A**) Brightfield and (**B**) H-E-stained images of full brain section utilized in confocal experiments, corresponding to the data shown in **Fig. 5**.

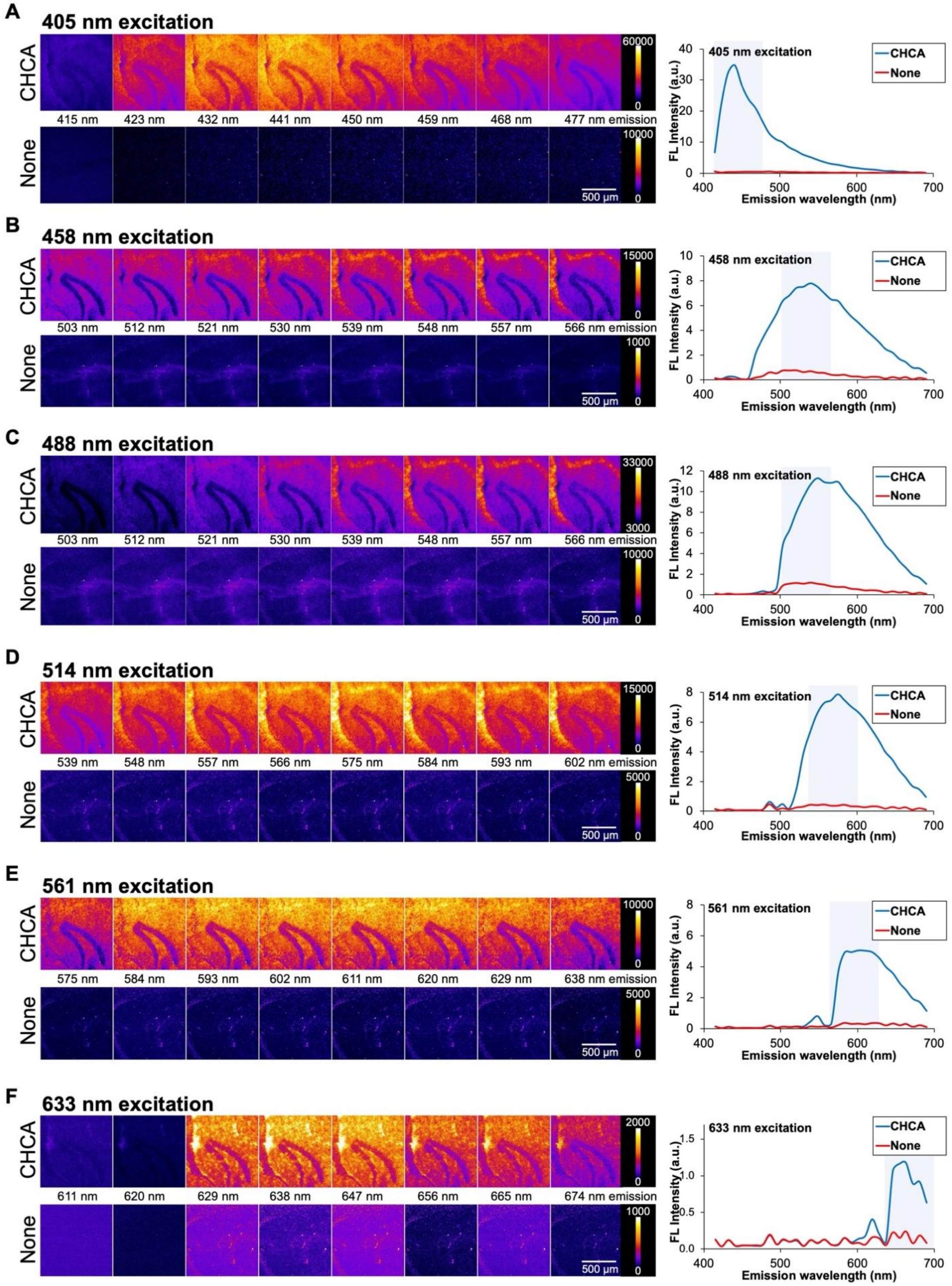

Figure S16. FluoMALDI pipeline using spectral imaging with laser-scanning confocal microscopy and MALDI MSI on mouse brain tissue sections half-coated with CHCA. Comparison of CHCA matrix-coated (top row, from blue box in **Fig. 5A**) *versus* uncoated (none, bottom row, from red box in **Fig. 5A**) hippocampal horn regions excited at (**A**) 405 nm, (**B**) 458 nm, (**C**) 488 nm, (**D**) 514 nm, (**E**) 561 nm, (**F**) 633 nm, and detected at various emission wavelengths ranging from 415 nm to 690 nm (from left to right), matching the blue highlighted regions in the corresponding spectral data (right).

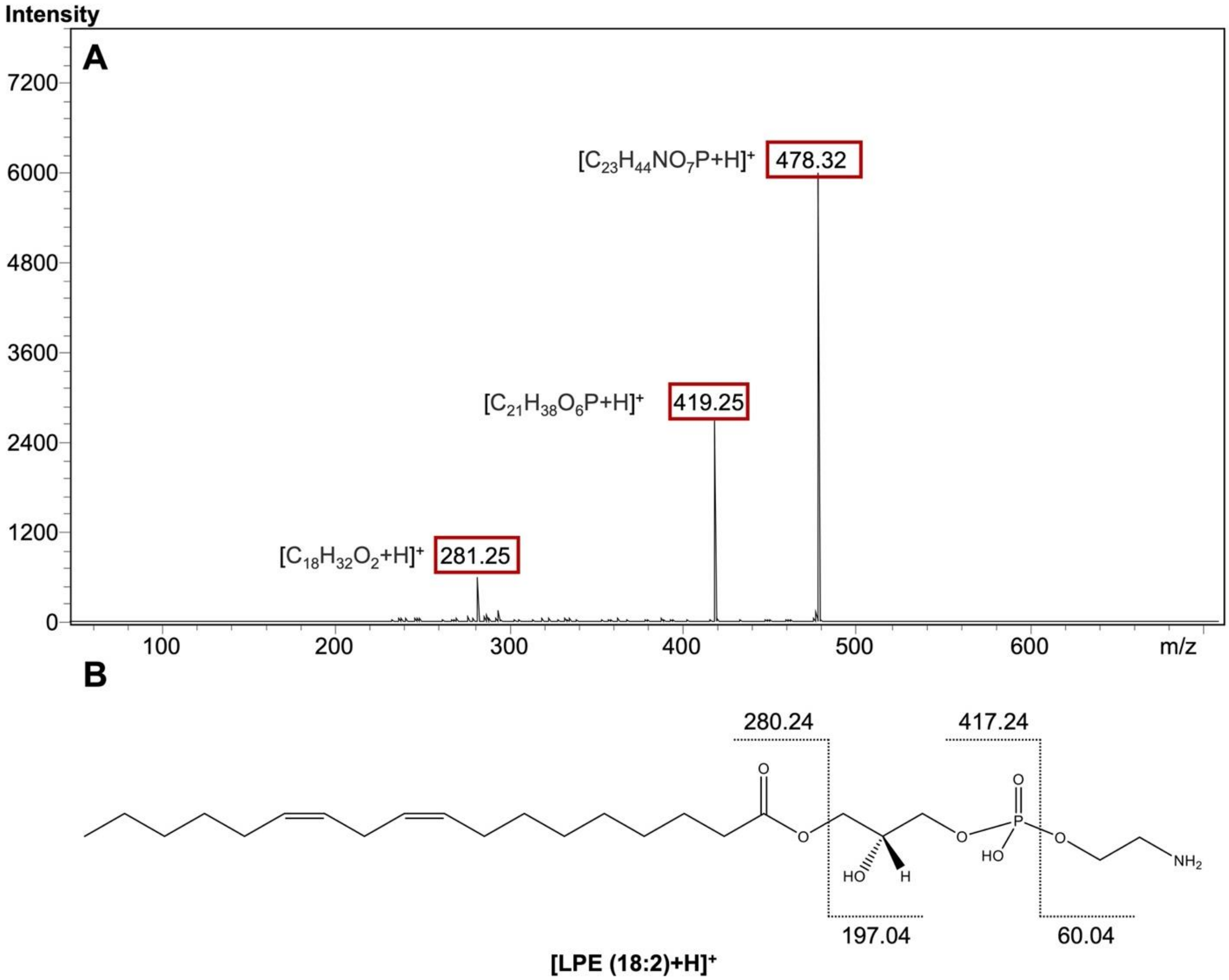

Figure S17. Positive ion mode tandem MS spectra of m/z$^+$ 478.33 Da identified as LPE (18:2), [M+H]$^+$.
(**A**) Precursor ion and fragmentation of m/z$^+$ 478.33 Da. (**B**) Chemical structure and fragmentation are shown based on characteristic fragments (boxed in red).

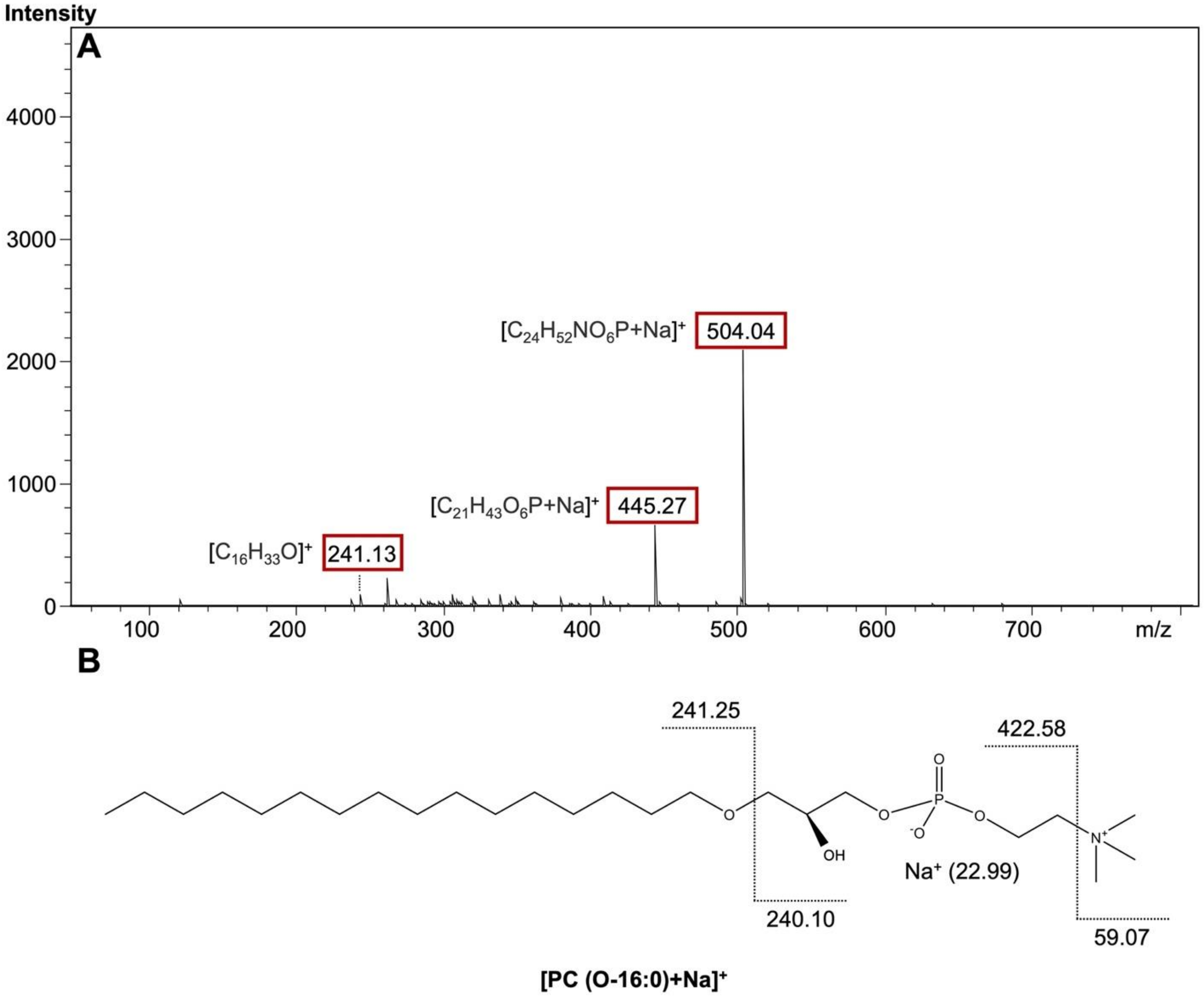

Figure S18. Positive ion mode tandem MS spectra of m/z$^+$ 504.04 Da identified as PC (O-16:0), [M+Na]$^+$.
(**A**) Precursor ion and fragmentation of m/z$^+$ 504.04 Da. (**B**) Chemical structure and fragmentation are shown based on characteristic fragments (boxed in red).

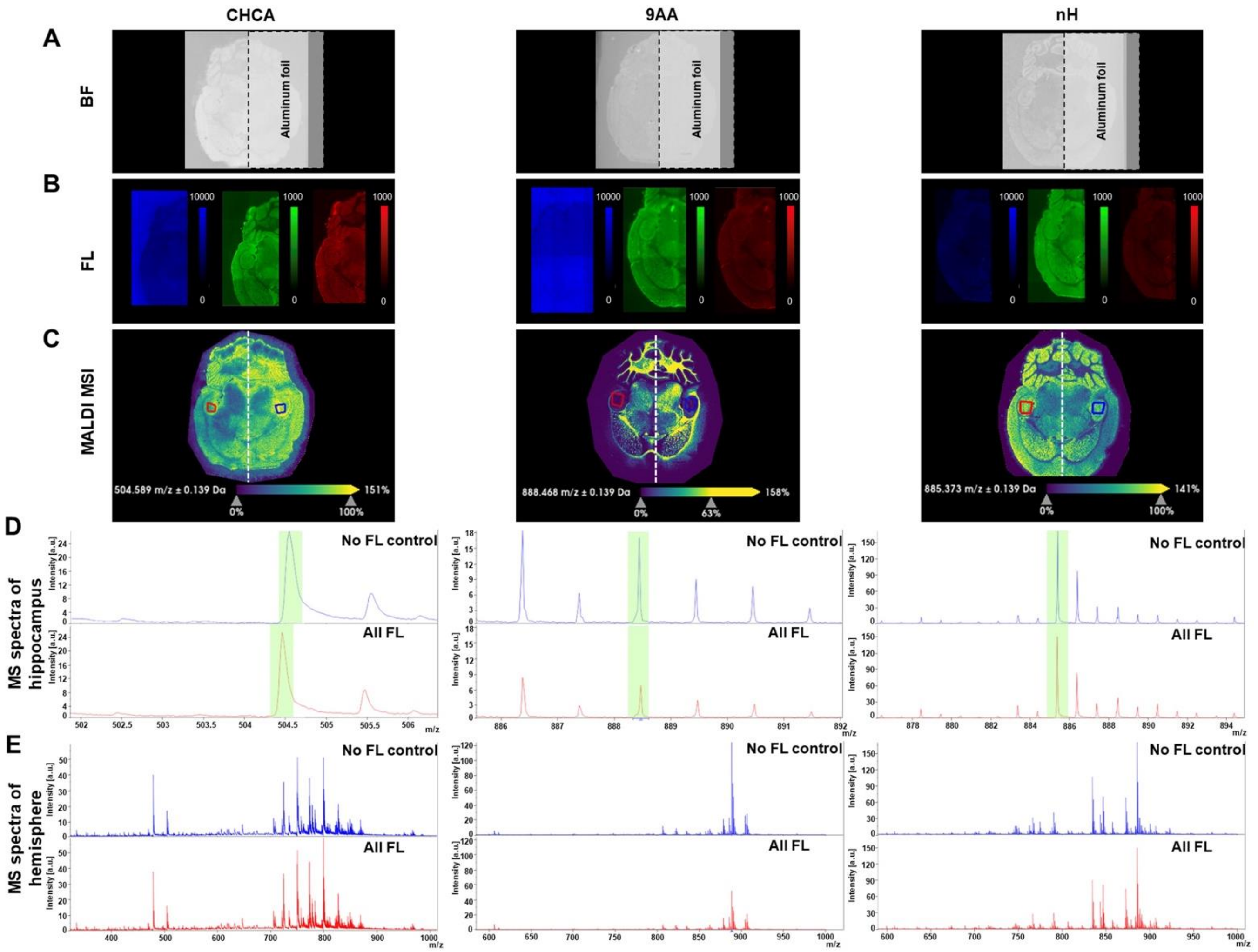

Figure S19. FluoMALDI imaging to examine the effects of fluorescence slide scanning on MALDI imaging. (**A**) Brightfield (BF), (**B**) blue, green, and red fluorescence (FL), and (**C**) MALDI MSI images for CHCA (left), 9AA (middle), and nH (right) matrix coating in which the right half of the mouse brain was blocked with aluminum foil during fluorescence slide scanning with three consecutive wavelength. Hippocampal regions are circled in red for the fluorescence-scanned left hemisphere and in blue for the no fluorescence control (right hemisphere) for spectral analysis. (**D**) Expanded average spectra comparing the blocked right hippocampus of no fluorescence control (blue) and the left fluorescence slide-scanned hippocampus (red). (**E**) Full average spectra of control right hemisphere shown in blue compared to fluorescence-scanned left hemisphere. The quantification of this spectral analysis is shown in Table S1.

Author Contributions

KG initiated, directed, and supervised the study. KG, SK, HWF, EY, and IB conceptualized the study. EY, XES, and THH carried out sample preparation. HWF, XES, LAR, and THH performed fluorescence microscopy experiments, data processing, and analysis. EY, CMT, XES, THH, DRB, and CCJ carried out MALDI MSI experiments, data processing, and analysis. MAS carried out single-crystal X-ray crystallography including data analysis and interpretation. EY, XES, DRB, CCJ, and CMT performed H-E staining and slide-scanning. EY, XES, THH, JHK, IB, SK, HWF, MAS, and KG designed the figures. EY, XES, and KG wrote the manuscript with support from HWF. All authors contributed to, provided critical feedback, reviewed, and approved the final manuscript.
